# Two-Stage Hierarchical Pruning (THP-CNN) of Convolutional Neural Networks for Rapid Pathogenic Bacterial Detection Using High-Resolution Colony Images in Intensive Care Units

**DOI:** 10.3390/diagnostics15182349

**Published:** 2025-09-16

**Authors:** Can Xie, Kefeng Li

**Affiliations:** Faculty of Applied Sciences, Macao Polytechnic University, Macao 999078, China; alexxiecan100@163.com

**Keywords:** THP-CNN, pathogenic bacterial detection, convolutional neural network compression, gut microbiota dysbiosis, precision medicine

## Abstract

**Background/Objectives**: Patients in Intensive Care Units (ICUs) have an elevated risk of infection. Accurate identification of pathogenic bacteria is critical for targeted interventions; however, convolutional neural networks (CNNs) face challenges of high computational demands and parameter redundancy. **Methods**: We developed a two-stage hierarchical pruning framework for CNN compression (THP-CNN), combining channel importance estimation with receptive field equivalence transformation for a 24-class pathogenic bacteria classification task. **Results**: THP-CNN (70% pruned) achieves an accuracy of 0.86 with 0.62 M parameters, outperforming ResNet-50 (0.72), MobileNet V2 (0.81), Inception (0.74), and AlexNet (0.62), with the 50% and 60% pruned variants in cross-validation stably maintaining a mean accuracy of 0.79. **Conclusions**: THP-CNN demonstrates potential for lightweight, real-time bacterial classification, offering a computationally efficient solution for automated pathogen detection.

## 1. Introduction

Intensive care unit (ICU) patients are at a heightened risk of infections, partly due to gut microbiota imbalances [1]. Accurate identification of pathogens is crucial for diagnosis and treatment. High-resolution classification of bacterial colonies, essential for morphological analysis, necessitates substantial computational resources [2].

This study employs a high-resolution bacterial colony dataset [3] comprising 24 clinically relevant pathogens, annotated to facilitate precise morphological differentiation. The dataset enables algorithmic development for automated pathogen detection in critical care, addressing the computational challenges associated with real-time classification in resource-constrained environments. However, CNNs [4,5,6,7] encounter significant obstacles when processing such complex imagery, primarily owing to high computational demands and parameter redundancy. This makes it difficult to achieve both real-time inference and high classification accuracy, potentially resulting in missed or erroneous detection of critical pathogens. For instance, despite its robust feature extraction capability, ResNet-50 [8] exhibits substantial parameter redundancy due to its deep architecture and fully connected layers, rendering it unsuitable for low-power embedded systems. MobileNet V2 [9,10,11] reduces parameter count via depthwise separable convolutions; however, its lightweight design may compromise the extraction of fine-grained local features necessary for distinguishing intricate colony morphologies. The Inception family of architecture enhances feature diversity through multi-scale convolutions, yet its branched structure frequently introduces redundant parameters, thereby increasing computational overhead. Although earlier models such as AlexNet [12,13] have fewer parameters, they lack sufficient representational capacity to capture subtle details in high-resolution colony images, leading to suboptimal classification performance.

To address these limitations, this study proposes a two-stage hierarchical pruning framework for CNN model compression [14]. The framework achieves a significant reduction in computational complexity while maintaining reliability in colony image recognition. This approach presents a novel framework for automatic and lightweight pathogen detection.

## 2. Materials and Methods

Conventional techniques for pruning superfluous parameters and accelerating inference in CNNs often face critical limitations:

- Imprecise assessment of kernel significance: Heuristic or statistical methods for quantifying the contribution of convolutional kernels to feature extraction frequently fail to distinguish between critical and non-critical components, potentially discarding essential information inadvertently [15,16].
- Overhead from auxiliary model training: Many approaches rely on auxiliary large-scale models to distill knowledge into compressed architectures. This dependency introduces additional computational and memory costs as training such models demands substantial time and resources, thereby inflating algorithmic complexity [17].
- Decoupled optimization of compression and acceleration: Traditional strategies typically address parameter pruning and inference speedup independently, neglecting the interplay between these objectives. This disjointed approach overlooks potential synergies between network compression and architectural simplification [18].

To address these challenges, the THP-CNN framework evaluates convolutional kernel importance through L2-norm quantification of the scaling parameters *γ* in batch normalization (BN) [19] layers. In contrast to conventional static pruning methods that depend on heuristic thresholds or dynamic pruning approaches that incur additional computational costs, THP-CNN innovatively integrates *γ* into the gradient computation during backpropagation. This mechanism facilitates an algorithm-driven decay process that progressively drives redundant kernel weights toward zero, thereby eliminating the subjectivity associated with manually defined thresholds. By leveraging the normalization properties of BN layers, THP-CNN preserves feature integrity after pruning without requiring extensive retraining, effectively mitigating the performance degradation observed in traditional structured pruning methods, such as filter pruning [20], which often arises due to dimensional mismatches.

Furthermore, THP-CNN introduces a principle of receptive field equivalence for convolutional layer fusion. This principle leverages the equivalence in spatial coverage between cascaded convolutional operations and a single extended convolution, enabling structural compression through the consolidation of sequential layers into a unified operation with an equivalent receptive field. The fusion reduces both network depth and parameter count while preserving representational capacity. In comparison to low-rank decomposition techniques such as singular value decomposition, the proposed approach avoids approximation errors inherent in matrix factorization and demonstrates effectiveness for chain-structured architectures including VGG. It enables reduction in network depth and departs from the narrow optimization pathway adopted by conventional lightweight models such as MobileNet, which are primarily based on depth-wise separable convolutions.

THP-CNN also adopts a lightweight design that does not rely on auxiliary components, thereby circumventing the limitations of knowledge distillation, which requires a separate teacher model, and dynamic pruning, which depends on specialized hardware support [21]. The method directly produces a compact and deployable model upon completion of pruning. Its core contribution resides in the integration of mathematically equivalent transformations into the model compression pipeline, enabling synergistic optimization of pruning and layer fusion. This allows for substantial parameter reduction while preserving the model’s feature representation capability.

**Figure 1:**
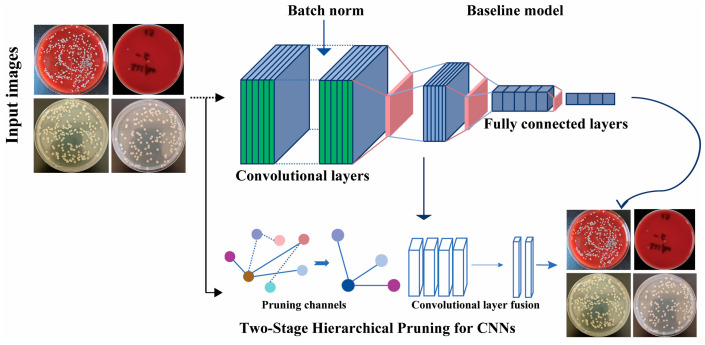
The THP-CNN framework. The sequential pipeline begins with a baseline model (comprising convolutional, batch normalization, and fully connected layers) performing feature extraction on input images. Subsequently, a two-stage hierarchical pruning strategy is implemented: (1) channel pruning reduces parameter redundancy, and (2) convolutional layer fusion enhances structural efficiency. The final output is a compact and high-performance model.

**Figure 2:**
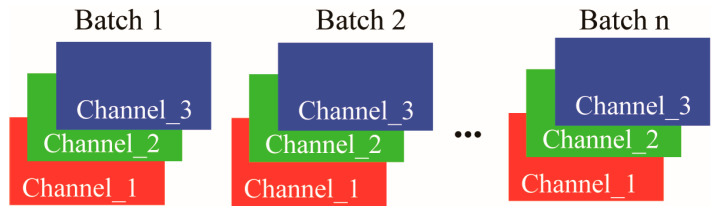
Operation of the BN layer. The diagram shows feature maps organized by batches and channels. For each channel, the BN layer normalizes the activations across all samples within a single batch.

The proposed methodology Figure 1 seamlessly integrates BN layers with convolutional operations, eliminating the necessity for additional parameters. By incorporating the L2 norm of the scaling factor γ into the training process, the model dynamically adjusts γ through gradient updates, enabling its progressive reduction across training epochs. This mechanism assigns implicit importance scores to convolutional kernels based on γ values, facilitating the systematic pruning of low-priority components. The retained kernels, deemed most salient, are prioritized for their sensitivity to feature extraction. Furthermore, the framework leverages receptive field equivalence Figure 2 to consolidate convolutional layers, achieving structural simplification without compromising model performance.

### 2.1. Dataset

We conducted evaluations using a publicly available dataset of bacterial colonies [3], specifically curated for deep-learning-based colony detection in clinical microbiology contexts. This dataset comprises 369 high-resolution digital images of 24 bacterial species relevant to infectious diseases, including pathogens commonly associated with infections in patients. Each image is annotated with precise bounding boxes around individual colonies, enabling accurate object detection and classification tasks. Notably, the dataset was acquired under non-standardized, real-world laboratory conditions using mobile phone cameras, with variations in lighting and background to enhance generalizability. While the dataset includes metadata specifying the background type for each image, it does not provide segmentation masks for non-colony regions such as the Petri dish walls or other artifacts. In our experiments, we leveraged the background information provided to minimize interference from non-biological elements during preprocessing.

### 2.2. Data Augmentation

Data preprocessing is crucial for ensuring the accuracy of experimental results and enhancing the generalization capability of models [22]. To ensure the transparency of our research and the reproducibility of our experiments, we systematically disclose the complete preprocessing pipeline applied to the original images. Prior to feeding the images into the model for training, initial screening and categorization were performed based on the metadata provided by the dataset (such as background color and Petri dish type) to exclude samples with abnormal imaging conditions or lower annotation quality.

**Figure 3:**
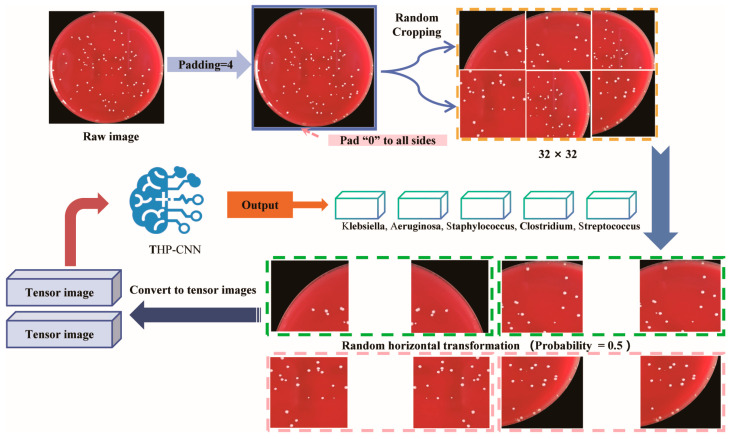
Data augmentation workflow. For the training set, images undergo a sequence of transformations including padding, random cropping, horizontal flipping (50% probability), and random rotation (±15 degrees). For the validation and test sets, only resizing is applied. All images are subsequently converted to tensors and normalized. This strategy expands the training data diversity while maintaining consistent evaluation conditions for the validation and test sets.

Subsequently, a series of standardization and data augmentation operations were applied to all retained images. Specifically, original images were resized to a fixed resolution to meet network input requirements, and normalization was conducted to adjust pixel value distributions to fit the numerical range required for model training. To further enhance the model’s adaptability to diverse morphologies and spatial distributions, various data augmentation strategies were introduced. These included random cropping to simulate colonies located in different regions of the field of view, thereby improving the model’s sensitivity to local features; horizontal and vertical flipping [20,23,24,25] to increase sample diversity and improve the model’s robustness to orientation invariance; and additional augmentations, such as random rotations, brightness adjustments, and contrast perturbations, to mimic the effects of uneven lighting or angular deviations encountered during actual image capture Figure 3.

### 2.3. Training Strategy

#### 2.3.1. Hardware Configuration

The system is equipped with 256 GB of memory. The CPU is an Intel Xeon Silver 4510 processor (Intel, Santa Clara, CA, USA), featuring two physical sockets with 12 cores per socket and 2 threads per core, resulting in a total of 48 logical CPU threads. It operates within a frequency range of 800 MHz (minimum) to 4.10 GHz (maximum) and is based on the x86_64 architecture, ensuring sufficient computational and scheduling capabilities on the CPU side during the training process. For GPU acceleration, the system integrates two NVIDIA RTX A6000 graphics cards (NVIDIA, Santa Clara, CA, USA), each with approximately 48 GB (49,140 MiB) of VRAM. The GPU driver version is 570.133.07, supporting CUDA 12.8.

#### 2.3.2. Hyperparameter Configuration

The experimental setup is configured as follows: the weight decay is set to $1\times 10^{-4}$, momentum is fixed at 0.9, the initial learning rate is 0.001, the batch size is 32, and the total training duration is 160 epochs. The learning rate is decayed by a factor of 10 at 80 and 120 epochs to facilitate convergence in later stages of training. For all models, the Mish [26] activation function is adopted, which has been shown to improve gradient flow and model performance through its smooth, non-monotonic properties. This configuration is consistently applied across all baseline and proposed models to ensure a fair comparison.

#### 2.3.3. Pruning Ratio

The determination of the pruning ratio depends on the original pre-trained CNN model, requiring the assessment of channel importance based on the weights of the pre-trained model. First, the parameters of all BN layers are extracted. During the pre-training phase, the weights are subjected to regularization decay involving the *γ* coefficient; thus, the magnitude of their absolute values reflects the importance of the corresponding channels, with larger absolute values indicating more significant contributions. Subsequently, the absolute values of the weights from every two consecutive BN layers are grouped together. Each group corresponds to two successive 3 × 3 convolutional layers, which are prepared for a subsequent equivalent merge into a 5 × 5 convolution. All such groups are then aggregated into a single global set. Finally, the absolute weight values within this global set are sorted in ascending order, and the pruning index is determined according to a predefined pruning ratio (e.g., 50%, 60%, or 70%), based on which pruning is executed.

### 2.4. Cross-Validation and Statistical Evaluation of Model Performance

For the classification tasks, we reported the best-fold metrics for all models Table 1. Additionally, all models were evaluated using cross-validation [27] Table 2, with each fold partitioned into training, validation, and test sets at a ratio of 6:2:2. The standard deviation (SD) [28] quantifies the variability of evaluation metrics—such as accuracy and precision—across different folds of cross-validation (i.e., across distinct data subsets). A smaller SD indicates that model performance is less sensitive to minor variations in data distribution and demonstrates greater stability. The 95% confidence interval (95% CI) defines the statistically estimated range within which the true model performance is expected to lie; a narrower interval reflects higher estimation precision and increased confidence in generalizing model performance to real-world scenarios. The combined use of SD and 95% CI enables a comprehensive assessment of model robustness and reliability, providing a more complete statistical foundation for evaluating model performance.

## 3. Results

This study evaluates the 24-class classification performance of the THP-CNN model (with pruning rates of 50%, 60%, and 70%) against four benchmark compact models—ResNet-50, MobileNet V2, Inception, and AlexNet—on an annotated dataset comprising 24 clinically relevant bacterial species (369 images; 56,865 annotated colonies). Cross-validation was employed to assess model stability, and the key findings are shown below.

### 3.1. Superiority of the THP-CNN Model

#### 3.1.1. Best-Fold Performance Comparison

- Regarding best-fold metrics, the THP-CNN series demonstrated significant advantages. The 70% pruned THP-CNN achieved the highest accuracy of 0.86, significantly outperforming MobileNet V2 (0.81), ResNet-50 (0.72), Inception (0.74), and AlexNet (0.62). The 70% pruned THP-CNN led with a precision of 0.85, surpassing the other models (0.61–0.82). The 60% pruned THP-CNN achieved a recall of 0.84, outperforming MobileNet V2 (0.81) and ResNet-50 (0.72). Both the 60% and 70% pruned THP-CNN models attained an F1 score of 0.82, outperforming MobileNet V2 (0.80) and the remaining models (0.59–0.74).
- Regarding model compactness, the parameter count of THP-CNN decreased substantially with an increasing pruning rate: the 70% pruned version had only 0.62 M parameters, which was considerably lower than ResNet-50 (23.56 M), Inception (21.83 M), AlexNet (57.10 M), and MobileNet V2 (2.25 M), demonstrating superior parameter efficiency.

#### 3.1.2. Cross-Validation Stability Analysis

- The cross-validation results (Mean ± SD, 95% CI) further confirm the overall superiority of THP-CNN. The 50% and 60% pruned THP-CNN models both achieved a mean accuracy of 0.79, which was significantly higher than ResNet-50 (0.69 ± 0.02), MobileNet V2 (0.76 ± 0.03), Inception (0.62 ± 0.11), and AlexNet (0.58 ± 0.02). Moreover, their narrower 95% confidence intervals (0.74–0.84) suggest more precise and consistent performance estimates compared to the wider interval of the more volatile Inception model (0.48–0.75).
- The mean trends for precision, recall, and F1 score align with those of accuracy: the 50% pruned THP-CNN achieved a precision of 0.77 ± 0.03, recall of 0.78 ± 0.04, and an F1 score of 0.75 ± 0.04, outperforming most baseline models. The 60% pruned THP-CNN showed a slight decrease in precision (0.75 ± 0.06) but maintained strong overall performance. Regarding model size, the THP-CNN variants exhibited significantly lower mean parameter counts (0.77–3.01 M) compared to the benchmark models, with low standard deviations (0.18–0.50), indicating controllable and consistent parameter reduction during pruning.

In sum, the THP-CNN model achieves a favorable balance between performance and efficiency in the 24-class bacterial colony classification task. Through its pruning strategy, it maintains or surpasses the classification accuracy of benchmark models while drastically reducing model size. Furthermore, the 50% and 60% pruned variants exhibit enhanced generalization stability, thereby providing a robust and efficient solution for the automated detection and classification of bacterial colonies.

### 3.2. Mathematical Modeling

To identify and prune superfluous convolutional kernels [26,27,28], we impose an L2 penalty on the scaling factors (*γ*) of the batch normalization (BN) layers associated with each kernel. This penalty is incorporated into the overall loss function during training. The magnitude of each *γ* coefficient thereby serves as a proxy for its corresponding kernel’s importance; kernels associated with *γ* values driven towards zero by the L2 regularization are deemed redundant and are subsequently pruned. The modified objective function is formulated as follows:

For a weight matrix $F\in R^{p}$, where $F=(f^{1},f^{2},\ldots ,f^{q})$, the L2 norm objective function is defined as follows:

(1) $$L_{reg}\left(F\right)=\frac{1}{n}\sum_{i=1}^{n}f^{i}+\frac{\mu }{2n}\sum_{j=1}^{q}{\left|\left|f^{j}\right|\right|}_{2}^{2}$$

The role of BN [21,29,30] in optimizing convolutional neural network training is well-documented. This study incorporates a post-convolutional normalization layer to stabilize the distributions of intermediate features. By mitigating intra-batch statistical variability and accelerating gradient propagation, this strategy improves the efficiency of model training. The significance of convolutional kernels is evaluated using the γ coefficients defined in Equation (2), which inform the selective elimination of filters with minimal contribution. The proposed pruning framework is illustrated in Figure 2, highlighting the integration of normalization metrics into the model compression pipeline:

(2) $$y=\frac{x-E[x]}{\sqrt{Var\left[x\right]+\varepsilon }}\gamma +b$$

Although the normalization step alleviates gradient saturation issues in part, it concurrently suppresses nonlinear representational capacity, thereby compromising model generalization. To overcome this constraint, the BN layer integrates an adaptive linear transformation post-whitening with the objective of reconstructing the inherent nonlinear properties of the input data. This strategy incorporates learnable parameters, *γ* (scaling coefficient) and *b* (offset parameter), to restore feature diversity and maintain expressive capacity.

This study proposes an approach to dynamically adjust the number of filters while preserving the effective receptive field dimensions in CNNs. The receptive field denotes the spatial extent in the input image that contributes to a specific feature map element at a given network layer. From the input perspective, it also characterizes the region where the CNN extracts discriminative patterns. Decreasing the filter count reduces the total multiplication–accumulation operations, thereby enhancing computational efficiency and accelerating inference speed. The pruned architecture retains filters essential for maintaining model accuracy, necessitating careful optimization to prevent performance degradation. To balance computational efficiency with feature representation fidelity, we utilize receptive field equivalence transformation methods to minimize redundant filters. The mathematical formulation for calculating receptive field size is as follows:

(3) $${RF}_{l}\;=\;{RF}_{l\;-\;1\;}+\;\left(k_{l}\;-\;1\right)\;\times \;\prod_{i\;=\;0}^{l\;-\;1}\;s_{i}$$

In this context, ${RF}_{l-1}$ denotes the spatial extent of the receptive field in the l−1 layer, $k_{l}$ represents the kernel size of the convolutional filter in the l-th layer, and $s_{i}$ corresponds to the stride value applied during convolution in the i-th layer. By exploiting the equivalence in receptive field characteristics between two sequential 3×3 convolutional operations and a unified 5×5 convolutional operation, the proposed approach facilitates architectural simplification through layer reduction.

## 4. Discussion

This study systematically compares THP-CNN (with pruning rates of 50%, 60%, and 70%) against ResNet-50, MobileNet V2, Inception, and AlexNet on a 24-class bacterial colony classification task. THP-CNN achieves significantly better balance between performance and parameter efficiency than baseline models, maintaining an accuracy of 0.86 at 70% pruning Table 1, with this advantage rooted in its unique structured pruning framework and network optimization logic.

THP-CNN employs a two-stage hierarchical pruning framework that quantifies convolutional kernel importance via the L2 norm of scaling parameters *γ* in batch normalization (BN) layers, integrating *γ* into gradient computation during backpropagation. This mechanism enables redundant kernel weights to naturally decay to zero during training, substantially mitigating the subjectivity associated with manual thresholds in traditional static pruning and eliminating the additional computational cost associated with dynamic pruning. Consequently, THP-CNN precisely identifies and retains the most salient convolutional kernels highly relevant to bacterial colony features (e.g., edge textures and color gradients) while eliminating noise-sensitive redundant parameters—explaining its high accuracy of 0.86 at 70% pruning.

In contrast, ResNet-50 (23.56 M parameters) mitigates vanishing gradients via residual connections but suffers from inherent redundancy in standard convolutional layers, resulting in lower accuracy (0.72) due to excessive feature channels, including noise irrelevant to colony classification. MobileNet V2 (2.25 M parameters) relies on depthwise separable convolutions for parameter compression, a strategy that may compromise the extraction of critical colony features. It yields an accuracy of 0.81—below that of THP-CNN’s 70% pruned model (0.86). THP-CNN innovatively introduces a receptive field fusion-based convolutional layer fusion principle: by leveraging the spatial equivalence between cascaded 3 × 3 convolutions and a single larger 5 × 5 convolution, consecutive layers are consolidated into unified operations with equivalent receptive fields. This reduces network depth and parameters to 0.62 M at 70% pruning—a value far lower than MobileNet V2′s 2.25 M—while avoiding approximation errors from low-rank decompositions (e.g., singular value decomposition), fully demonstrating “precise compression rather than uniform simplification”.

Inception theoretically accommodates morphological diversity via parallel multi-scale convolutions but exhibits high sensitivity to data variability (accuracy: 62 ± 11%; 95% CI: 0.48–0.75), whereas THP-CNN leverages BN layer normalization to maintain feature integrity after pruning, requiring minimal retraining and ensuring stable generalization (95% CI: 0.74–0.84) with less performance fluctuation. AlexNet (57.10 M parameters; accuracy: 0.62) lacks feature reuse mechanisms due to simple layer stacking, highlighting the limitations of traditional architectures in fine-grained feature extraction.

THP-CNN achieves extreme parameter reduction (0.62–3.01 M) without auxiliary components (e.g., teacher models or specialized hardware), using *γ*-guided kernel se-lection and receptive field fusion to precisely capture subtle colony features. Moderate pruning (50% and 60%) maintains a mean accuracy of 0.79 in cross-validation, confirming that *γ*-guided decay preserves core feature channels. However, 70% pruning shows reduced stability (95% CI: 0.59–0.88), validating that pruning prioritizes feature preservation over minimal parameter count.

This study’s dataset comprises 369 images and 56,865 annotated colonies, representing a relatively limited scale that may constrain the learning of rare morphologies. To mitigate overfitting, various data augmentation techniques (e.g., rotation and brightness adjustment) were implemented. Future work will explore joint training with multiple bacterial colony datasets to enhance sample diversity. Rigorous testing demonstrates that THP-CNN’s lightweight design is particularly suitable for small-data scenarios.

In conclusion, THP-CNN establishes a highly efficient framework for bacterial colony classification through *γ*-driven dynamic pruning, receptive field fusion-based structural optimization, and dependency-free lightweight design. It achieves extreme parameter reduction while preserving key features, demonstrating significant advantages over ResNet-50 (residual connections), MobileNet V2 (uniform compression), Inception (multi-scale sensitivity), and AlexNet (traditional stacking), even under limited data conditions. It is thus a generalizable paradigm for lightweight model design in microbiological image classification.

## 5. Conclusions

This study proposes THP-CNN, a structured model compression framework that integrates channel importance estimation with receptive field transformation via layer fusion. The framework achieves an optimal efficiency–accuracy trade-off: at 60% pruning, THP-CNN attains an accuracy of 0.85 with 1.19 million parameters, outperforming all four baseline CNN models; at 70% pruning, it maintains a high accuracy of 0.86 with only 0.62 million parameters, demonstrating exceptional compression efficiency. THP-CNN eliminates the reliance on manual thresholding in structured pruning through a *γ*-guided dynamic decay mechanism during training, which adaptively suppresses redundant convolutional kernels while preserving essential feature representations. This approach resolves inherent dimensional mismatches in conventional pruning methods and ensures structural coherence in the pruned network. The mechanism effectively removes noise-sensitive parameters while retaining critical morphological features such as edge textures and color gradients; this efficacy is evidenced by the model’s high accuracy even under aggressive pruning. Experimental results confirm the effectiveness of THP-CNN in enabling efficient model compression for resource-constrained inference. By seamlessly integrating importance quantification and structural optimization, this work explores a new avenue for structured pruning, providing insights into creating generalizable, lightweight models for microbiological image classification.

## Figures and Tables

**Table 1:** Comparison of best-fold performance metrics for the THP-CNN and benchmark models on the colony dataset.

Models	Accuracy	Precision	Recall	F1 Score	Parameters (M)
ResNet-50	0.72	0.76	0.72	0.71	23.56
MobileNet V2	0.81	0.82	0.81	0.80	2.25
Inception	0.74	0.79	0.74	0.74	21.83
AlexNet	0.62	0.61	0.62	0.59	57.10
THP-CNN (50% pruned)	**0.8** **5**	**0.** **79**	**0.8** **3**	**0.8** **0**	**2.70**
THP-CNN (60% pruned)	**0.8** **5**	**0.8** **1**	**0.8** **4**	**0.8** **2**	**1.** **19**
THP-CNN (70% pruned)	**0.** **8** **6**	**0.8** **5**	**0.8** **4**	**0.** **8** **2**	**0.** **62**

**Table 2:** Cross-validation performance of the THP-CNN and benchmark models on the colony dataset, reported as mean ± SD (95% CI).

Models	Accuracy	Precision	Recall	F1 Score	Parameters (M)
	Mean ± SD, 95% CI				
ResNet-50	0.69 ± 0.02 (0.66–0.72)	0.72 ± 0.03 (0.69–0.76)	0.69 ± 0.02 (0.66–0.72)	0.67 ± 0.03 (0.64–0.70)	23.56 ± 0.00 (23.56–23.56)
MobileNet V2	0.76 ± 0.03 (0.71–0.80)	0.76 ± 0.04 (0.72–0.81)	0.76 ± 0.03 (0.71–0.80)	0.74 ± 0.04 (0.69–0.79)	2.25 ± 0.00 (2.25–2.25)
Inception	0.62 ± 0.11 (0.48–0.75)	0.61 ± 0.13 (0.45–0.77)	0.62 ± 0.11 (0.48–0.75)	0.59 ± 0.12 (0.44–0.73)	21.83 ± 0.00 (21.83–21.83)
AlexNet	0.58 ± 0.02 (0.56–0.61)	0.59 ± 0.03 (0.54–0.63)	0.58 ± 0.02 (0.56–0.61)	0.55 ± 0.03 (0.52–0.59)	57.10 ± 0.00 (57.10–57.10)
THP-CNN (50% pruned)	**0.79 ± 0.03 (0.75–0.84)**	**0.77 ±** **0.03 (0.72–0.81)**	**0.78 ±** **0.04 (0.73–0.82)**	**0.75 ±** **0.04 (0.71–0.80)**	**3.01 ±** **0.36 (2.57–3.46)**
THP-CNN (60% pruned)	**0.79 ±** **0.04 (0.74–0.84)**	**0.75 ±** **0.06 (0.67–0.83)**	**0.77 ±** **0.05 (0.70–0.83)**	**0.74 ±** **0.06 (0.66–0.81)**	**1.57 ±** **0.50 (0.96–2.18)**
THP-CNN (70% pruned)	**0.74 ±** **0.12 (0.59–0.88)**	**0.69 ±** **0.16 (0.49–0.89)**	**0.70 ±** **0.14 (0.53–0.88)**	**0.68 ±** **0.15 (0.48–0.87)**	**0.77 ±** **0.18 (0.55–0.99)**

## Data Availability

The original data presented in the study are openly available in https://figshare.com/articles/dataset/Annotated_dataset_for_deep-learning-based_bacterial_colony_detection/22022540/3 (accessed on 10 August 2025). The code has been made publicly available on GitHub and can be accessed at: https://github.com/Li-OmicsLab-MPU/THP-CNN.git (accessed on 10 August 2025).

## Abbreviations

CNN	Convolutional Neural Network
THP-CNN	Two-Stage Hierarchical Pruning CNN method
ICU	Intensive Care Unit
BN	Batch Normalization
95% CI	95% Confidence Interval
SD	Standard Deviation
